# Supplementary material for: Recombinant Toxoplasma gondii Ribosomal Protein P2 Modulates the Functions of Murine Macrophages In Vitro and Provides Immunity against Acute Toxoplasmosis In Vivo
Source: Vaccines (Basel). 2021 Apr 7;9(4):357. doi: 10.3390/vaccines9040357 (PMC8068005; doi:10.3390/vaccines9040357)
Supplement: Supplementary file 1 [file vaccines-09-00357-s001.pdf]

vaccines

MDPI

Article

Recombinant *Toxoplasma Gondii* Ribosomal Protein P2 Modulates the Functions of Murine Macrophages In Vitro and Provides Immunity against Acute Toxoplasmosis In Vivo

ZhengQing Yu[1], YuJia Lu[1], ZhaoYi Liu[1], Muhammad Tahir Aleem[1], JunLong Liu[2], JianXun Luo[2], RuoFeng Yan[1], LiXin Xu[1], XiaoKai Song[1] and XiangRui Li[1*]

[1] MOE Joint International Research Laboratory of Animal Health and Food Safety, College of Veterinary Medicine, Nanjing Agricultural University, Nanjing 210000, China; 2018207044@njau.edu.cn (Z.Y.); 11118310@njau.edu.cn (Y.L.); 17118306@njau.edu.cn (Z.L.); 2018207076@njau.edu.cn (M.T.A); yanruofeng@njau.edu.cn (R.Y.); xulixin@njau.edu.cn (L.X.); songxiaokai@njau.edu.cn (X.S.)

[2] State Key Laboratory of Veterinary Etiological Biology, Key Laboratory of Veterinary Parasitology of Gansu Province, Lanzhou Veterinary Research Institute, Chinese Academy of Agricultural Sciences, Lanzhou 730046, China; liujunlong@caas.cn (J.L.); luojianxun@caas.cn (J.L.)

* Correspondence: lixiangrui@njau.edu.cn; Tel.: +86-025-84399000

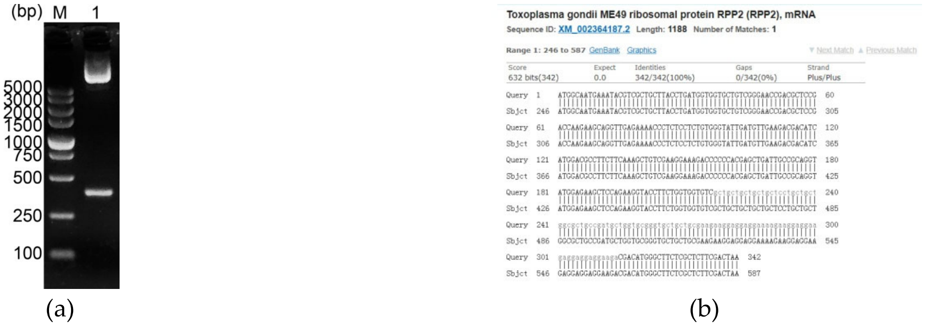

Figure S1. The results of double digestion and sequence alignment. (**a**) Double digestion analysis of recombinant plasmid pET32a/TgRPP2. Lane M: DNA molecular weight marker DL5000; Line 1: pET32a/TgRPP2 plasmid after double digestion. (**b**) Sequence alignment results of the recombinant plasmid pET32a/RPP2. Compared with the nucleotide sequences of RPP2 gene (Genbank: XM_002364187), sequence analysis was con-ducted through the Blast program online (http://www.blast.ncbi.nlm.nih.gov/blast.cgi).

Table S1. Determination of total IgG in the sera of the immunized mice in days 0, 7, and 14. Results were evaluated using one-way ANOVA analysis followed by Dunnett's test and shown as the mean of the OD450 ± standard deviation.

Day	Group	Total IgG (OD values)	*P* value	*P* value
0	Blank	0.681 ± 0.063	-	0.8677 [b]
	Control	0.663 ± 0.057	0.8677 [a]	-
	rTgRPP2	0.690 ± 0.049	0.9612 [a]	0.7276 [b]
	Blank	0.670 ± 0.054	-	0.9065 [d]
7	Control	0.692 ± 0.073	0.9065 [c]	-
	rTgRPP2	0.984 ± 0.113	0.0004 [c]	0.0007 [d]
	Blank	0.740 ± 0.083	-	0.8500 [f]
14	Control	0.770 ± 0.065	0.8500 [e]	-
	rTgRPP2	1.188 ± 0.109	< 0.0001 [e]	< 0.0001 [f]

[a], [b], [c], [d], [e], and [f] were compared with the blank group at days 0, the control group at days 0, the blank group at days 7, the control group at days 7, the blank group at days 14, and control group at days 14, respectively.

Table S2. Determination of subclasses IgG1, and IgG2a in the sera of the immunized mice in days 0, 7, and 14. Results were evaluated using one-way ANOVA analysis followed by Dunnett's test and shown as the mean of the OD450 ± standard deviation.

Day	Group	IgG1 (OD values)	*P* value	*P* value	IgG2a (OD values)	*P* value	*P* value
0	Blank	0.670 ± 0.051	-	0.8437 [b]	0.691 ± 0.062	-	0.8517 [b]
	Control	0.648 ± 0.055	0.8437 [a]	-	0.711 ± 0.051	0.8517 [a]	-
	rTgRPP2	0.662 ± 0.082	0.9768 [a]	0.9327 [b]	0.679 ± 0.066	0.9439 [a]	0.6767 [b]
7	Blank	0.657 ± 0.046	-	0.9607 [d]	0.809 ± 0.058	-	0.8563 [d]
	Control	0.668 ± 0.059	0.9607 [c]	-	0.784 ± 0.061	0.8563 [c]	-
	rTgRPP2	0.855 ± 0.086	0.0022 [c]	0.0034 [d]	1.001 ± 0.099	0.0071 [c]	0.0031 [d]
14	Blank	0.711 ± 0.056	-	0.9225 [f]	0.807 ± 0.044	-	0.7347 [f]
	Control	0.735 ± 0.079	0.9225 [e]	-	0.845 ± 0.070	0.7347 [e]	-
	rTgRPP2	0.996 ± 0.148	0.0037 [e]	0.0068 [f]	1.104 ± 0.111	0.0004 [e]	0.0013 [f]

[a], [b], [c], [d], [e], and [f] were compared with the blank group at days 0, the control group at days 0, the blank group at days 7, the control group at days 7, the blank group at days 14, and control group at days 14, respectively.
